# Chemical Profile, Antioxidant Capacity, and Antimicrobial Activity of Essential Oils Extracted from Three Different Varieties (Moldoveanca 4, Vis Magic 10, and Alba 7) of *Lavandula angustifolia*

**DOI:** 10.3390/molecules26144381

**Published:** 2021-07-20

**Authors:** Mihaela Alexandra Bogdan, Simona Bungau, Delia Mirela Tit, Dana Carmen Zaha, Aurelia Cristina Nechifor, Tapan Behl, Dorina Chambre, Andreea Ioana Lupitu, Lucian Copolovici, Dana Maria Copolovici

**Affiliations:** 1 Doctoral School of Biomedical Sciences, University of Oradea, 410087 Oradea, Romania; mihaela.alexandra.bogdan@gmail.com (M.A.B.); mirela_tit@yahoo.com (D.M.T.); 2 Department of Pharmacy, Faculty of Medicine and Pharmacy, University of Oradea, 410028 Oradea, Romania; 3 Department of Preclinical Disciplines, Faculty of Medicine and Pharmacy of Oradea, University of Oradea, 410073 Oradea, Romania; danaczaha@gmail.com; 4 Clinical Emergency Hospital of Oradea, 410169 Oradea, Romania; 5 Analytical Chemistry and Environmental Engineering Department, University Politehnica of Bucharest, 011061 Bucharest, Romania; aureliacristinanechifor@gmail.com; 6 Department of Pharmacology, Chitkara College of Pharmacy, Chitkara University, Punjab 140401, India; tapanbehl31@gmail.com; 7 Faculty of Food Engineering, Tourism and Environmental Protection, “Aurel Vlaicu” University of Arad, 310330 Arad, Romania; dorinachambree@yahoo.com (D.C.); lucian.copolovici@uav.ro (L.C.); dana.copolovici@uav.ro (D.M.C.); 8 Institute for Research, Development and Innovation in Technical and Natural Sciences, “Aurel Vlaicu” University of Arad, 310330 Arad, Romania; pag.andreea@yahoo.com

**Keywords:** *Lavandula angustifolia*, essential oils, antimicrobial activities, antioxidant capacity, geranium, tea tree

## Abstract

Chemical composition, antioxidant capacity, and antimicrobial activity of lavender essential oils (LEOs) extracted from three different varieties of *Lavandula angustifolia* Mill. (1-Moldoveanca 4, 2-Vis magic 10, and 3-Alba 7) have been determined. These plants previously patented in the Republic of Moldova were cultivated in an organic agriculture system in the northeastern part of Romania and then harvested in 3 consecutive years (2017–2019) to obtain the essential oils. From the inflorescences in the complete flowering stage, the LEOs were extracted by hydrodistillation. Then, their composition was analyzed by gas chromatography coupled with mass spectrometry (GC-MS) and by Fourier Transformed Infrared spectroscopy (FT-IR). The major identified constituents are as follows: linalool (1: 32.19–46.83%; 2: 29.93–30.97%; 3: 31.97–33.77%), linalyl acetate (1: 17.70–35.18%; 2: 27.55–37.13%; 3: 28.03–35.32%), and terpinen-4-ol (1: 3.63–7.70%; 2: 3.06–7.16%; 3: 3.10–6.53%). The antioxidant capacity as determined by ABTS and DPPH assays indicates inhibition, with the highest activity obtained for LEO *var.* Alba 7 from 2019. The in vitro antimicrobial activities of the LEOs and combinations were investigated as well, by using the disk diffusion method and minimum inhibitory concentration (MIC) against the Gram-positive bacterial strain *Staphylococcus aureus* (ATCC 6538), Gram-negative *Pseudomonas aeruginosa* (ATCC 27858), *Escherichia coli* (ATCC 25922), the yeast *Candida albicans* (ATCC 10231), and clinical isolates. Our results have shown that LEOs obtained from the three studied varieties of *L. angustifolia* manifest significant bactericidal effects against tested microorganisms (*Staphylococcus aureus* and *Escherichia coli*), and antifungal effects against *Candida albicans*. The mixture of LEOs (*Var.* Alba 7) and geranium, respectively, in tea tree EOs, in different ratios, showed a significant enhancement of the antibacterial effect against all the studied strains, except *Pseudomonas aeruginosa*.

## 1. Introduction

Essential oils (EOs) are defined as a concentrated natural plant-derived mixture of volatile biomolecules that could be extracted from diverse plant organs [1]. Initially, when they were discovered, they were used for therapeutic purposes (as medicines), and later in the perfume and cosmetics industry and as cleaning materials, respectively, for food and beverages [1], and are reported to have various beneficial effects on human health (antibacterial, antifungal, antioxidant, antiparasitic, antiseptic, antiviral, etc.) and also insecticidal activities [2,3,4,5,6].

After more than a century of using antibiotic therapy, studies have shown an increase in resistance to antimicrobial drugs, both in Gram-negative and Gram-positive pathogens; thus, some infections became untreatable, resulting in increased morbidity/mortality in all countries [7,8]. Moreover, the pathogens are increasingly resistant to common antibiotics, making the therapy management more difficult and impedes optimal treatment of patients [7,8]. Consequently, the interest in the identification and development of natural alternative antibacterial agents is growing.

The different plant organs of plants (root, leaves, bark, flowers, fruits, seeds, resin) contain EOs that can be easily extracted and purified by various procedures: hydrodistillation, solvent extraction, steam distillation, supercritical fluid extraction [9]. EO of lavender (LEO) is one of the most popular EO known that can be extracted from several varieties of this plant; moreover, four significant varieties of lavender must be mentioned as the most relevant for this plant, namely, *Lavandula angustifolia* (recognized as the commercial lavender and being the most extensively cultivated), *Lavandula latifolia*, *Lavandula stoechas*, and *Lavandula x intermedia* (which is a sterile cross between *L. angustifolia* and *L. latifolia*) [10]. All EOs obtained from different varieties of lavender are volatile, being composed of aromatic substances, more precisely mixtures (synthesized by plants organs) of secondary metabolites mainly comprising groups of correlated biosynthetic compounds (i.e., C10–C15 terpene, known as isoprene derivatives, aromatics, and/or aliphatic compounds, having low molecular weight) which are giving the characteristic smell (aroma) of this plant [11].

The variable content of linalool (in the concentration of 20–45%) and linalyl acetate (in the concentration of 25–47%) were determined as the major components found in the EO of *L. angustifolia*, which also contains modest concentrations of other characteristic chemical compounds (i.e., eugenol (1,8-cineol), lavandulyl acetate, lavandulol, terpinen-4-ol) and small amounts of α-pinene, α-terpineol, β-borneol, camphor, caryophyllene, geraniol, limonene, nerol, etc. [12].

The quality and composition of EOs vary widely and are determined by many factors, including the variety of the plant, cultivation conditions (cultivation area, soil properties, weather and climatic conditions, altitude, harvest period, etc.), and the procedure used to obtain the EO (extraction with an organic solvent, extraction of the supercritical fluid/liquid using carbon dioxide, maceration, percolation, steam or fractional distillation, etc.) [11].

Two compounds of lavender EO (namely, linalool and linalyl acetate) have been determined as being the main substances that enhance the antimicrobial activity against food bacteria (*Escherichia coli*, *Enterobacter cloacae*) [13]. Other EO compounds (namely, α- and β-pinenes, limonene) manifest antibacterial action against various pathogenic bacteria in humans [14], the in vitro effect of EO against vancomycin-resistant *Enterococcus faecium* and methicillin-resistant *Staphylococcus aureus* also being observed [15]. Increased antibacterial activity can be attributed to the presence (in a large percentage) of oxygenated monoterpenes; they destroy the cell morphology and viability of the biofilm by increasing the permeability and reducing the polarization of the cytoplasmic membrane. Free hydroxyl (–OH) groups can release protons, leading to conformational changes and ultimately cell death [16].

Published data have shown that certain EOs have an antimicrobial activity that can sometimes depend on one or two of its main constituents; often, the ratio of these main active substances is not the only determining factor for the actions of EOs; however, the interactions between these major compounds and the minor constituents of EOs are undoubtedly extremely important [17]. Studies in which binary/ternary combinations were tested and verified highlighted different synergistic antimicrobial activities for various compounds, respective fractions, of EO [18,19]. Moreover, there are published data on the synergistic effect of combinations between different EOs, due to the correlated activities of two or more chemical compounds contained in these EOs. This may result in a potentiation of fungistatic activity (highly advantageous in the pre/post-harvest protection), as pathogens cannot easily acquire resistance to more compounds of two/more EOs [20].

Our research evaluated the chemical composition and antioxidant capacity of LEOs obtained from three different varieties of *L. angustifolia* (Moldoveanca 4, Vis magic 10, and Alba 7) originated and patented in the Republic of Moldova [21,22,23], and cultivated in an organic agriculture system in the northeastern part of Romania, in 3 harvesting years (2017–2019). Moreover, the antimicrobial activity determinations were performed on each individual LEO and on LEO combined with geranium and tea tree EOs, with proven antimicrobial activity, to find possible positive interactions of them and to obtain combinations of oils with superior antimicrobial properties/action.

## 2. Results

### 2.1. Chemical Composition of L. angustifolia, var. Moldoveanca 4, Alba 7, and Vis Magic 10 Essential Oils Determined by GC-MS

The specific chemotype, for each variety, in each of the 3 years of harvesting (2017–2019) is presented in Table 1. All analyses were performed in triplicate.

### 2.2. FT-IR Analyses

**Figure 1:**
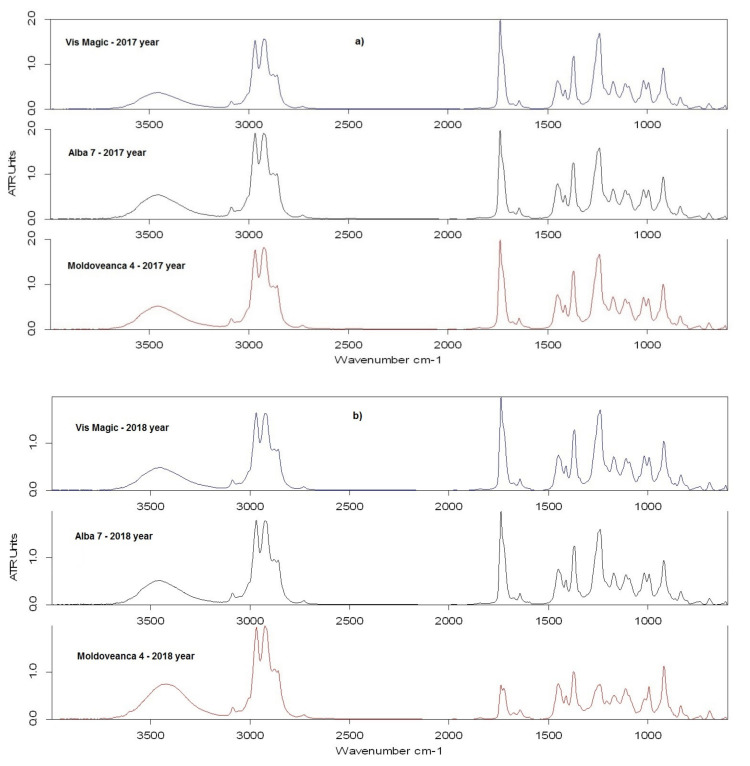

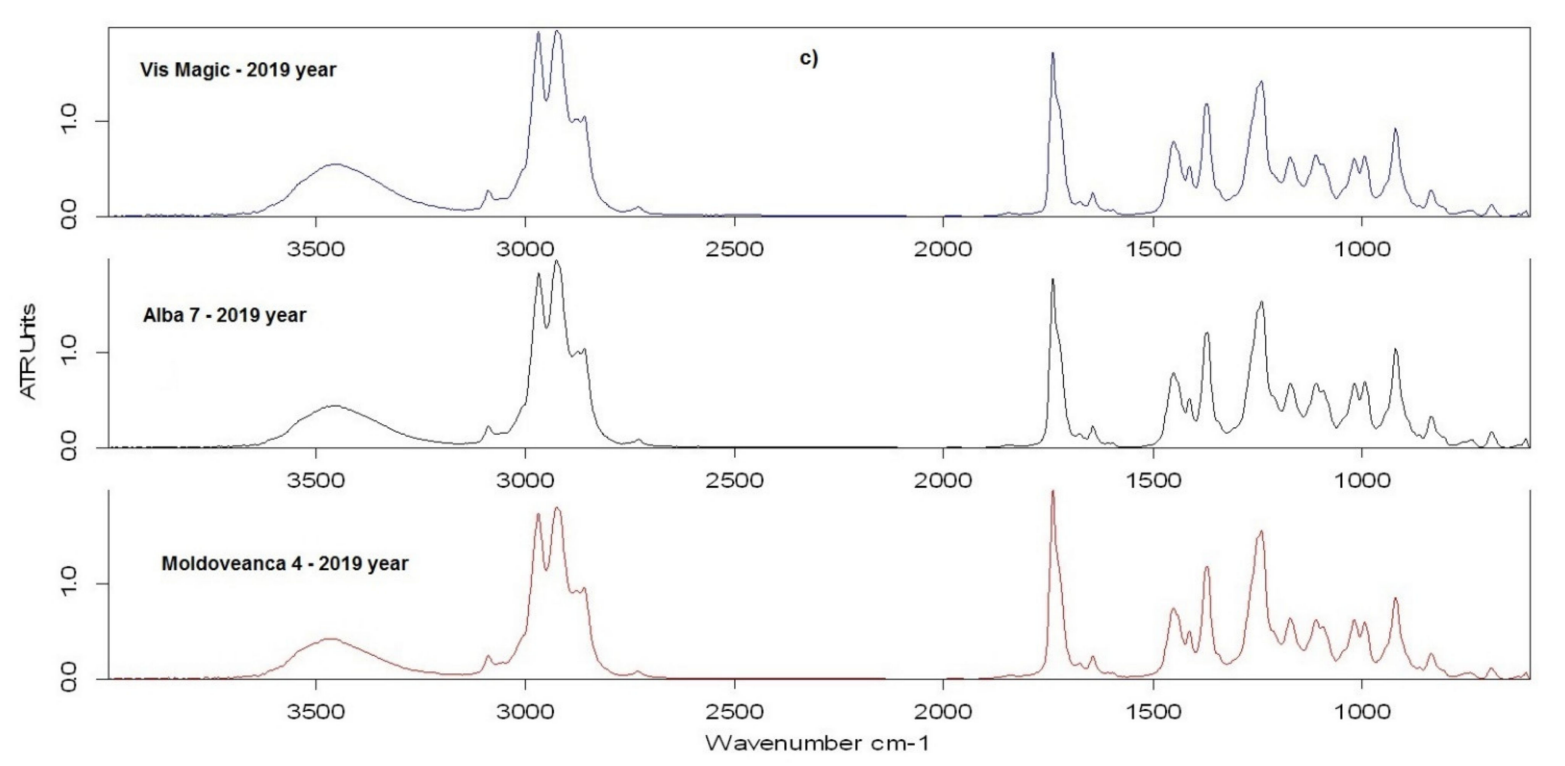
ATR-FTIR spectra for the essential oil samples from the investigated varieties obtained in 2017 (**a**), 2018 (**b**), and 2019 (**c**), respectively.

The obtained ATR-FTIR spectra for the LEOs are depicted in Figure 1. The wavelengths values for recorded bands on the 600–4000 cm^−1^ range and the vibration assignment, carried out using literature data, are detailed in Table 2. The ATR-FTIR spectra (Figure 1) obtained for all investigated LEO samples present a very good similarity over the entire wavenumbers scanned range, except the Moldoveanca 4 obtained in 2018 (1800–900 cm^−1^ range).

According to the results depicted in Figure 1a–c and summarized in Table 2, the most important bands recorded in all recorded spectra are at: ~3455 cm^−1^, 1738 cm^−1^, 1239 cm^−1^, ~993 cm^−1^, and 919 cm^−1^, being characteristic for functional groups in aliphatic esters and secondary alcohols. In the *Var.* Moldoveanca 4 (1), 2018 year spectrum, the characteristic band located at 1111 cm^−1^ is better highlighted because of ν_sym_ (C–O) vibration from alcohols.

**Figure 2:**
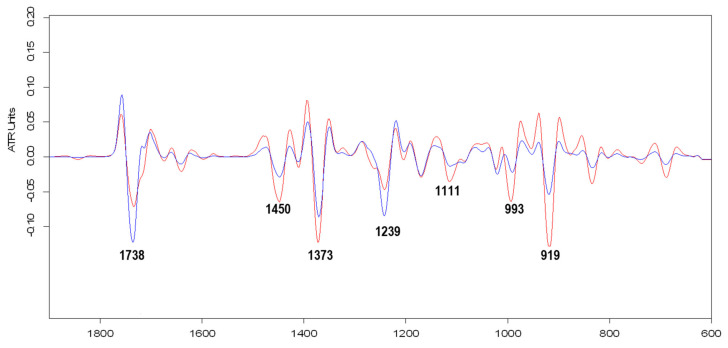
The second derivative spectra for the *Var.* Moldoveanca 4 (1), 2018 year (red), and *Var.* Vis magic 10 (3), 2017 year, (blue) LEOs samples.

The second derivative spectra, obtained by using the Savitzky–Golay algorithm on 600–1900 cm^−1^ wavenumbers range, for *Var.* Moldoveanca 4 (1), 2018 year and *Var.* Vis magic 10 (3), 2017 year samples are presented in Figure 2.

The differences in the chemical composition of the *Var.* Moldoveanca 4, 2018 year, and *Var.* Vis magic 10, 2017 year, samples led to important changes in the intensities of the characteristic signals for esters (1738 cm^−1^ and 1239 cm^−1^) and alcohols (1111 cm^−1^, 993 cm^−1^, and 919 cm^−1^).

### 2.3. Assessment of the Antioxidant Activity

The antioxidant activity of the samples determined by ABTS assay and radical scavenging DPPH (1,1-diphenyl-2-picrylhydrazyl) free assay indicated that the inhibition is varying from 56.20 to 76.04% for ABTS and from 32.37 to 69.83% for DPPH, depending on the variety and year of harvesting of the plants (Table 3).

### 2.4. Assessment of Antimicrobial Effects

Evaluation of antimicrobial activity in *Staphylococcus aureus* shows significant inhibition of bacterial growth, with similar results in the reference strain *Staphylococcus aureus* (ATCC 25923) and the clinical isolates strain (Table 4). *Var.* Vis magic 10 showed the highest activity against *Staphylococcus aureus* when the three varieties used were compared; the strongest inhibitory effects were obtained when combinations with tea tree EO and geranium EO were used (the diameters of the inhibition zone exceeding those of antibiotics used as positive controls). *Var.* Vis magic 10 had significantly better action on the reference strain, and the combination Alba 7:tea tree acted significantly better on the isolated strain. All tests were performed in triplicates, on each reference strains and those clinically isolated from patients.

LEOs also showed significant antibacterial activity against *Escherichia coli. Var.* Vis magic 10 showed the largest diameter (25.5 ± 0.70 mm) of the inhibition zone against *E. coli.* The combinations of lavender:geranium EOs at a 2:1 ratio (*v*/*v*) inhibited significantly more (*p* = 0.02) the isolated strains, as shown in Table 5. The combinations of lavender:tea tree, in the ratio of 1:1 and 2:1, showed complete inhibition of bacterial growth, as it is summarized in Table 2. All tests were performed in triplicates, on each reference strain and those clinically isolated from patients.

None of the tested samples, both individually and in mixtures, inhibited the growth of *Pseudomonas aeruginosa*, regardless of the inoculum used (0.10, 0.25, and 0.5 Mc Farland unit). Evaluation of the antifungal activity of lavender’s variety on *Candida albicans*, on the reference strain ATCC 10231 and on the clinical isolates one showed a total inhibition in the case of the combinations lavender + tea tree and lavender + geranium, when a 0.5 unit Mac Farlane inoculum was used for testing. For this reason, the antifungal effect of a Mac Farlane 1-unit inoculum was prepared and tested. Considering a 12 mm threshold as the diameter of the inhibition zone, an inhibitory effect on *Candida albicans* growth was also found when the three lavender varieties were tested, without statistically significant differences (*p* > 0.05) between the action on the wild strain and the one on the reference strain (Table 6), as well as a total inhibition of fungal growth in the presence of lavender + tea tree and lavender + geranium combinations. All tests were performed in triplicates, on each reference strain and those clinically isolated from patients.

Var. Vis magic 10 showed the most potent antibacterial effect when LEOs were compared as such, with no statistically significant differences (*p* < 0.05) compared to the other varieties. The combinations of lavender and geranium (samples 4 and 5), respective to those with tea tree (6 and 7), in different proportions, showed a significant increase of the antibacterial effect on all the studied strains, except *Pseudomonas aeruginosa*. The ratio of oils in the case of the combination with tea tree did not lead to significant changes in the antibacterial action of the mixture, the average diameter of the inhibition zone being equal in the case of both ratios (1:1 and 2:1).

All oil samples presented inhibitory action on bacterial strains at different dilutions, except for *P. aeruginosa*. The MIC values for tested strains ranged from 0.78 to 25 µg/mL, and it seems that clinical isolates presented MIC values slightly larger than ATCC ones. Antimicrobial action on *S. aureus* and *E. coli* were similar (1.56 µg/mL). *C. albicans* was inhibited even at 1:128 dilution corresponding to 0.78 µg/mL. Table 7 presents the MIC values with inhibitory action on several microorganism strains (*S. aureus*, *E. coli*, and *C. albicans*) for both clinical strains and ATCC. All tests were performed in triplicates, on both each reference strain and those clinically isolated from patients.

## 3. Discussion

Studying different possibilities to create new antimicrobial products to treat infections with bacteria resistant to drugs has gained increasing attention lately, determined by antibiotic-resistant bacteria growth and the absence of new antibiotics available on the market [7,8]. The suggested approaches comprise identifying options to antibiotics as well as identifying adjuvants [24]. Considering the antibacterial properties of EOs [2,3,4], they may help decrease bacterial resistance [25,26].

The chemical composition of LEOs fluctuates according to variety and pedoclimatic conditions and influences their action [11,27,28]. The complete chemical composition of the mentioned LEOs (**1**—LEO *Var.* Moldoveanca 4; **2**—LEO *Var.* Alba 7; **3**—LEO *Var.* Vis magic 10) obtained in this study, in 3 consecutive years (2017, 2018, 2019), was determined by GC-MS. The high amount of main compounds, namely: linalool (>30%) and linalyl acetate (>27%, expecting sample from 2018, which is 17.7%), and the small amount of terpinen-4-ol (<8%) found in all LEOs samples, are indicating a good quality of the LEOs, according to other published data [29,30]. Camphor was found in small amount in the samples (in the range 0.12% for **3** in 2019, and 0.98% for **2** in 2019) values that are < 1.0% as the standard is proposing to be for LEO, while for 1,8-cineole, the percentage was found to range from 0.77% for **3** in 2017 to 3.15% for **1** in 2018, the latest value being slightly higher than that proposed in the standard (3%) [31].

The major constituents of EOs can represent up to 85%, whereas other components are present in trace amounts [32]. *Var.* Moldoveanca 4 has the highest amount of linalool and terpinen-4-ol and the lowest amount of linalyl acetate, while *Var*. Vis magic 10 has the highest amount of linalyl acetate and the lowest amount of linalool and terpinene-4-ol. The EOs’ action is mainly given by the synergic action of the contained chemical compounds, though there are multiple evidences of a single chemical compound exerting antimicrobial activity individually [18,19,33,34].

Attenuated Total Reflectance—Infrared Fourier Transform (ATR-FTIR) spectroscopy was used to complete the data obtained by the other presented methods and to investigate the influence of differences in the chemical composition of LEO samples on their spectral characteristics (peak wavelength and relative absorbance to the main FTIR band values). Except for the LEO *Var.* Moldoveanca 4 obtained in 2018 (Figure 1a, in red), the ATR-FTIR spectra obtained for all other investigated LEOs samples (Figure 1a–c) are very similar to those presented in the literature by Lafhal et al. [35], and Samfira et al. [36]. Correlating the data from Table 2 with those obtained from the GC-MS analysis (Table 1), it can be seen that the components which are in higher concentration in samples (linalool, terpinen-4-ol, linalyl acetate, lavandulyl acetate, caryophyllene, ocimene) dominate the resulting vibrational spectra of LEOs. In contrast, the components at low concentration do not have a significant influence. Similar behavior has been reported in the literature for other EOs extracted from various herbs [37,38].

The broad band specific for H-bonded hydroxyl compounds (stretching vibration of O-H) can be observed in all LEOs spectra at ~3455 cm^−1^. In the case of the **1**—*Var.* Moldoveanca 4 – 2018 sample, this band is more intense due to the higher content of linalool and terpinen-4-ol. Bands recorded in the 3000–2700 cm^−1^ range characterize the asymmetrical and symmetrical stretching vibrations of the C-H bond, and the band located at 3088 cm^−1^ indicates the presence of the double C=C bond in compounds from LEOs [39,40]. These bands are not discriminatory and do not provide useful information for quantification. The same observation is regarding the bands located at 1450 cm^−1^, ~1412 cm^−1,^ and ~1370 cm^−1^ due to the bending vibration of the C-H bond from methyl and methylene groups [35,39].

Based on data reported by Lafhal et al. [35] for the main pure compounds of lavender essential oil, the following assignments of the recorded bands for investigated LEOs could be made: the bands located at ~1738 cm^−1^ (stretching vibration of carbonyl C=O from ester groups); 1644 cm^−1^ and ~1595 cm^−1^ (stretching vibration of C=C); 1239 cm^−1^, 1171 cm^−1^ and ~1018 cm^−1^ (stretching vibration of C-O from linalyl acetate) are specific for linalyl acetate and lavandulin acetate; the bands located at ~1207 cm^−1^ (O-H bending); 1111 cm^−1^ (stretching C-O from secondary alcohols); 993 cm^−1^ and 919 cm^−1^ are specific for linalool and terpinen-4-ol; the bands recorded at 1674 cm^−1^, ~863 cm^−1^ (wagging vibration form caryophyllene), 834 cm^−1^ (wagging vibration form occimene), ~739 cm^−1^ and ~690 cm^−1^ (*cis*-C-H out-of-plane bending from ocimene) for caryophyllene and ocimene [35].

In the case of the spectrum for **1**-LEO *Var.* Moldoveanca 4—2018 year, some changes can be observed due to the sample’s different chemical composition compared to the other LEOs’ analyzed samples. Thus, the band located at 1738 cm^−1^ (stretching vibration of carbonyl C=O from ester) decreases in intensity, and a second peak appears at 1723 cm^−1^ (Figure 1 and Figure 2). Additionally, the band recorded at 1239 cm^−1^ decreases in intensity due to the low content of esters (linalyl acetate + lavandulyl acetate = 18.07%, Table 1).

At the same time, a new weak band was recorded at 1207 cm^−1^, and significant increases in the intensity of the 1111 cm^−1^, 994, and 918 cm^−1^ bands, specific to linalool and terpinen-4-ol were observed in accordance with the high content of secondary alcohols from sample **1**. This behavior suggests that the chemical composition of the samples influences the bands’ intensity of the functional.

By correlating the spectral data with those obtained from the GC-MS analysis (Table 1), it can be seen that the components which are in higher concentration in LEOs samples (linalool, terpinen-4-ol, linalyl acetate, lavandulyl acetate, caryophyllene, ocimene) dominate the resulting vibrational spectra. Similar behavior has been reported in the literature for other Eos extracted from various herbs [37,38].

The antioxidant capacity of the LEO was evaluated by two different methods, as follows: DPPH radical-scavenging activity and ABTS radicals scavenging activity. The ABTS assay is more sensitive than DPPH to identify the antioxidant capacity since it has faster reaction kinetics and a higher response to antioxidant molecules. The results lead to a high percent inhibition, from 56.20 to 76.04%, depending on the variety, the climatic conditions, and year of plant harvesting [41]. The values of the percent inhibition, as determined by the ABTS assay, are not statistically significantly different for the LEOs harvested from the same variety of the plant (Table 4). As regarding the data obtained by using the DPPH assay, the inhibition % was in the domain from 32.37% (LEO *Var.* Alba 7 obtained in 2019) to 69.83% (LEO *Var.* Vis Magis obtained in 2017). The analyzed LEOs, due to their composition, from which we determined 38 compounds, showed good inhibitory activity against ABTS radical than the DPPH radical, similar to that reported by Kıvrak for six LEOs obtained from cultivars harvested in Turkey [29].

EOs that contain monoterpene hydrocarbons, oxygenated monoterpenes, and/or sesquiterpenes have elevated antioxidative properties [42], which is due to a few found components (α and β-pinene, camphene, *p*-cymene-8-ol, limonene, ocimene, and terpinene) in the analyzed LEOs. Consequently, LEOs’ antioxidant activity can be attributed to the same aforementioned chemical compounds. Found in these LEOs, mono—and sesquiterpene hydrocarbons, and oxygenated monoterpenes as well—have radical scavenging action [43]. The findings of a published study, which considered antioxidant activity and evaluated 98 pure EOs in this regard, showed that monoterpene hydrocarbons had a major influence on this fact [44], and also demonstrated that EOs that are rich in non-phenolic compounds that have remarkable antioxidant activity [45].

The minor inhibition % was recorded for LEO *var*. Alba 7 was obtained in 2019 (56.20%); therefore, we have chosen to continue our antimicrobial studies on LEO obtained in 2019.

EOs proved efficiency in many antimicrobials pathways due to their complex composition. The action of the main chemical components of LEOs (linalyl acetate, linalool, and terpinen-4-ol) was studied, revealing their activity mechanism directed towards damaging the cell membrane lipid layer, determining bacterial cell leakage [46,47,48]. Both the EO type and the microorganisms strain taken into study determine the antimicrobial activity mechanism. Gram-positive bacteria are known to be more sensitive to EOs than Gram-negative bacteria [49,50]. This is possibly because Gram-negative bacteria have a rigid outer membrane, are more complex, rich in lipopolysaccharide (LPS), restricting the diffusion of the hydrophobic compound through it. In contrast, Gram-positive bacteria do not present this membrane, being surrounded by a thick peptidoglycan wall whose density permits the access of small antimicrobial molecules towards the cell membrane [51]. Favored by the lipophilic ends of lipoteichoic acid existing in the cell membrane, Gram-positive bacteria facilitate the hydrophobic compounds of EOs intrusion [52].

The present study assessed the LEOs antimicrobial action on significant Gram-positive and Gram-negative pathogens in humans. *S. aureus* generates infection of the skin, and other infections, which can be severe in healthcare settings (such as pneumonia, osteomyelitis, endocarditis bacteremia, or sepsis). *E. coli* (a Gram-negative bacteria present in the environment, human, and animal intestines) can provoke respiratory infections, urinary infections, pneumonia, diarrhea, etc. *P. aeruginosa* leads to pneumonia, blood infections, or post-surgical infections. The yeast *C. albicans* affects mucosal areas and local systemic infections in the case of immune malfunction. Except for *P. aeruginosa*, all microorganisms used were sensitive to the tested EOs. *P. aeruginosa* has a higher intrinsic resistance to antimicrobials, which is partly due to its low outer membrane permeability, and it does not possess diffusion porins [53].

The antibacterial effect of *Var.* Vis magic 10 EO is clearly superior to other LEO varieties studied, and the most intense antibacterial effect is against *E. coli*, followed by *S. aureus* and *C. albicans*, a fact that may be explained by the sensitivity of the type of bacterium, the composition, type, and harvesting period of the studied plant material [54]; additionally, the concentration and the intraspecific, seasonal variation of the EO’s composition must be considered [55].

The antimicrobial activity of the LEOs was demonstrated even with 1/32 dilutions, as MIC determination revealed (Table 7). The weakest antibacterial effect was registered for LEO *Var.* Alba 7, but in combination with tea tree EO and geranium EO, its antibacterial effect was potentiated. In various previous studies, focusing on combination therapy with LEOs, increased attention has been paid to synergistic interactions due to the use of multidirectional antimicrobial activity, resulting in a marked decrease in toxicity and increased efficacy of LEOs [56,57]. LEOs’ antimicrobial effects can be potentiated when using combinations, as follows: between various components of LEOs; LEOs along with other EOs; LEOs and other antimicrobial compounds. In the present study, we chose to test the combination of LEO: geranium and LEO: tea tree because so far, the activity of both oils against antibiotic-resistant bacteria enjoys considerable interest, so that methicillin-resistant *Staphylococcus aureus* (MRSA) receives the greatest attention [46,58].

The mixtures have superior action on all studied strains compared to individual species, both on the positive controls and against the clinical isolates. In *S. aureus*, the strongest inhibitory effects were obtained when combinations with tea tree and geranium EOs were used, the diameters of the inhibition zones exceeding those of antibiotics such as Vancomycin. Additionally, the effect on the isolated strain was more substantial in combination with tea tree EO; its inhibition was significantly higher than that on the reference strain. *E. coli* strains were completely inhibited by the combination of LEO with tea tree EO. Total inhibition was also produced against *E. coli* strains when all combinations were tested.

The superior antibacterial action of the *Var.* Alba 7 LEO, observed both in combination with geranium/tea tree EOs, may result from the EOs chemical elements’ possible interactions. To overcome antimicrobial resistance, the mechanism of action must be elucidated when diverse combinations of EOs mixtures are used [59]. Furthermore, the synergistic effects of LEO in combination with other EOs have been demonstrated in previous studies [60]. Associating specific oils generates synergism due to the combined action of some EOs elements, especially by their major compounds, the minor compounds also influencing the favorable interactions noticed [20].

## 4. Materials and Methods

### 4.1. Obtaining Essential Oils

The aerial parts of *Lavandula angustifolia* Mill., Moldoveanca 4, Alba 7, and Vis magic 10 varieties (that are protected by Moldova Patents variety of plants (73MD, 2010; 74MD, 2010; 75MD, 2010) [21,22,23], were harvested in 2017, 2018, and 2019, respectively, from an organic culture in a farm, located in Curtești village, Botoșani County, Romania (geographical coordinates 47°41′51″ N 26°38′53″ E). LEOs were obtained from the aerial organ plants were harvested manually in the second half of June. These varieties are resistant to droughts, frost, and wintering, being suitable for cultivation in the pedoclimatic conditions of Romania. It is worth mentioning that Moldoveanca 4 is an early cultivated variety, unlike Alba 7 and Vis magic 10.

LEOs were obtained in the hydrodistillation station of the cultivator ECOLAND PRODUCTION SRL, Romania, from freshly harvested plant material, then packaged in dark glass bottles (amber color) and stored at +4 °C. Hydrodistillation was chosen because it leads to a high yield of LEO, being the main known method, ultra-verified, and used to obtain LEO immediately after harvesting the plants; it was performed by the steam-distillation process, about 1 h, at a constant temperature of 100 °C. All the LEOs samples were freshly analyzed in the harvesting year.

### 4.2. Determination of the Chemical Composition of Lavender Essential Oils by GC-MS

The constituents of lavender oils were determined by a gas chromatography method, using a gas chromatograph (GC) (Shimadzu2010, Kyoto, Japonia) coupled with a triple quadruple mass spectrometer (MS) (TQ 8040, Shimadzu, Kyoto, Japan. The column used was of Optima 1MS (30 m × 0.25 mm i.d., the film thickness of 0.25 mm, (Macherey-Nagel, Duren, Germany, with helium as carrier gas and a flow rate of 1 mL/min.

The oven temperature was started at 70 °C for 11 min and raised to 190 °C (at a rate of 5 °C/min) and then at 240 °C (at a rate of 20 °C/min), where it was left for 5 min. The injector and MS source temperatures were set at 250 °C and 200 °C, respectively. The injection volume was one μL sample with a split ratio of 10:1. The compounds from the analyzed samples were identified based on their mass spectra using the NIST 14 and Wiley 09 mass spectrum libraries (Scientific Instrument Services, Palmer, MA, USA) and compared with that from ISO 3515:2002 [61]. All analyses were performed in triplicate.

### 4.3. FT-IR Analysis

ATR-FTIR spectra of LEOs were recorded on 600 and 4000 cm^−1^ wavelength range using a Bruker Vertex 70 (Bruker Corporation, Bremen, Germany) Spectrometer equipped with a Pike Miracle ATR device. For each measurement, a sample volume of ~10 μL was placed directly on the surface of the ZnSe ATR crystal in the Teflon depression and covered with a metal cover pressed with the upper handle of the device to avoid evaporation. The experimental spectrophotometric data were recorded with a resolution of 4 cm^−1^. For each sample of lavender essential oil, the spectra were obtained in triplicate, and the average spectrum of three measurements with 32 scans (3 × 32 spectra/sample) was depicted. Prior to every ATR measurement, the ZnSe crystal was carefully cleaned with isopropyl alcohol, and an air background spectrum was performed.

Spectra were recorded without any sample preparation, and OPUS 6.5 software (Bruker) was employed for data acquisition, normalization, and baseline correction as well as to evaluate the relative absorbance values of the recorded FTIR bands.

### 4.4. Determination of the Antioxidant Activity by ABTS Assay

The antioxidant capacity of LEOs was determined using the scavenging activity of ABTS * radical, following a previously reported method [62] with slight modifications. The ABTS * reagent stock solution was prepared by mixing equal quantities of ABTS reagent and 2.45 mm aqueous solution of sodium persulfate. The mixture was allowed to react at room temperature overnight. To analyze the scavenging activity of the samples, 1 mL ABTS * solution was mixed with 0.5 mL LEO sample. The control was obtained using ultrapure water. After 10 min of incubation time in the dark, the absorbance was recorded using a UV-VIS spectrophotometer (Specord 200, Analytik Jena, AG, Jena, Germany) at λ = 734 nm and a 10 mm quartz cuvette. Different Trolox standard concentrations were used (0.025–1.0 mM, Figure S1). All experiments were performed in triplicates, and the results were expressed as mmol trolox equivalent antioxidant capacities (TEAC)/L and % inhibition.

### 4.5. Determination of the Antioxidant Activity by DPPH Assay

The antioxidant capacity of the LEOs was evaluated by using the DPPH assay, as reported earlier [63]. A total of 0.1 mL sample was mixed with 3 mL of 0.2 mm ethanolic DPPH• solution. After 60 min of incubation in the dark at room temperature, the absorbance was recorded using a UV-VIS spectrophotometer (Specord 200 from Analytik Jena, AG, Jena, Germany) at λ = 517 nm and a 10 mm quartz cuvette. As a reference, positive controls containing 0.02–4.0 mm Trolox were prepared (Figure S2). All experiments were completed in triplicates, and the results were expressed as % inhibition and mg Trolox/L.

### 4.6. Preparation of Bacterial Strains

The LEOs antimicrobial activity obtained from all three varieties (Moldoveanca 4, Alba 7, and Vis magic 10) of *L. angustifolia* was performed on several Gram-positive and Gram-negative microorganisms (reference strains and strains isolated from patients) and yeast. These microorganisms were chosen given their frequency in the etiology of human infections, including fungi: *Staphylococcus aureus* ATCC 25923, *Escherichia coli* ATCC 25922, *Pseudomonas aeruginosa* ATCC 27853, and *Candida albicans* ATCC 10231.

SC Sanimed International Impex SRL, Bucharest Romania, provided freeze-dried reference strains in the form of pellets. When using them, with sterile forceps, in aseptic conditions, each lyophilized strain from the vial was extracted and inserted into a tube containing 5–7 mL of liquid culture medium (Tryptic Soya Broth). The tubes were incubated at 35 ± 2 °C for 3–5 h. Then, the contents of the tubes were gently shaken, and a drop of the suspension was inoculated on a solid culture medium distributed in the Petri dish. Sowing was completed with a loop by making streaks to develop isolated colonies. *Staphylococcus aureus*, *Escherichia coli*, and *Pseudomonas aeruginosa* were sown on agar Columbia + 5% ram’s blood, and *Candida albicans* on Sabouraud Glucose Agar. *Escherichia coli* and *Pseudomonas aeruginosa* were also grown on Levine agar medium (BioMaxima SA, Lublin, Poland).

The same strains and respective culture media were used to prepare fresh cultures obtained from isolates from patients. After inoculation, the plates were incubated at 35 ± 2 °C for 24–48 h. They tested three clinical isolates, from different patients, with different positives samples but with the same phenotype as reference strain. All strains used were from new cultures not exceeding 24 h.

### 4.7. Testing of Antimicrobial Activity

#### 4.7.1. Diffusion Method

The preparation and handling of inoculation suspensions, inoculated plates, and antimicrobial discs affect the size of the diameter of the inhibition zone and therefore require careful preparation. To determine the zones of inhibition, the diffusion method was used on the Mueller–Hinton Agar medium for bacteria and Sabouraud Glucose Agar for *Candida albicans*. It was prepared according to the manufacturer’s instructions (BioMaxima, Lublin, Poland).

The microbial inoculum was prepared in saline solution with a density of 0.5 McFarland for bacteria and 1 McFarland for fungi, using the McFarland densitometer (DensiCHEK Plus from bioMerieux, Inc., Durham, NC, USA). This inoculum was spread using a sterile cotton swab evenly over the entire surface of the agar plate to obtain uniform growth.

Approximately 6 mm filter paper discs were impregnated with EOs by depositing 10 μL of undiluted LEO of each lavender variety and combinations with geranium and tea tree in 1:1 and 1:2 ratios, which were coded as follows: Moldoveanca 4—sample no. **1**; Vis magic 10 —sample no. **2**; Alba 7—sample no. **3**; Alba 7: geranium 1:1—sample no. **4**; Alba 7:geranium 2:1—sample no. **5**; Alba 7:tea tree 1:1—sample no. **6**; Alba 7:tea tree 2:1—sample no. **7**.

The prepared discs were placed on the Petri dishes inoculated with the microorganisms to be studied at distances of at least 15 mm from the edge of the plate and 30 mm between the centers of two neighboring disks, using sterile forceps for a maximum 15 min. To perform the quality control, antibiotics from different classes, respectively antifungals recommended and interpreted according to CLSI 2020 [64], were tested in the same conditions on the same plate (Table 8).

Plates containing the inoculum were incubated at 35 ± 2 °C for 18 ± 2 h for bacteria and 72 h for *Candida albicans*. After incubation, the zones of inhibition were read at the point where no obvious growth is detected by the naked eye.

#### 4.7.2. Microdilution Method

The Minimal Inhibitory Concentration (MIC) value is the lowest concentration of an antibacterial agent that prevents bacterial growth in optimal conditions (24 h of incubation for bacteria and 48 h for *Candida albicans* at 37 °C). The MIC values were calculated using the micro-dilution broth method, starting with 10 mL graded doses (*v*/*v*) of different samples of oil diluted in acetone (Merck KGaA, Damstadt, Germany). These solutions were mixed with a 100 μL Mueller–Hinton broth to obtain concentrations from 0.78 to 25 μL/mL for oils. An inoculum containing 1 × 10^6^ colony forming units (CFU)/mL per well was added to broth with various oil concentrations to 96-well microtiter plates. MIC was established after incubation at 37 °C under aerobic conditions by estimation of the visible growth of a microorganism. Negative controls were included in each assay to determine the antimicrobial activity of the solvent, and culture controls were included to confirm sterility and viability. All tests were performed in triplicates, on each reference strain and those clinically isolated from patients.

### 4.8. Statistical Analysis

Data were processed by one-way ANOVA via GraphPad Prism (version 5.0 for Windows, GraphPad Software, San Diego, CA, USA). Data among the same species harvested in different years for a specific test (e.g., DPPH assay, ABTS assay) were compared with ANOVA. Then, Tukey’s Multiple Comparison Test determined statistically significant differences, and means labeled with different letters for F values at *p* < 0.05 were considered statistically significant.

## 5. Conclusions

Based on the present obtained results, it can be concluded that lavender essential oil harvested from three varieties of *Lavandula angustifolia* Mill in 3 years is richest in linalyl acetate (more than 27.55%), which is one of the main components with antimicrobial activity, and in linalool (more than 29.93%), a component that is desired in cosmetics. In addition to the main bioactive compounds, the analyzed essential oils are rich in terpinen-4-ol (3.06–7.70%), trans-beta-ocimene (1.52–6.99%), *cis*-beta-ocimene (0.43–2.70%), and caryophylene (3.55–5.39%), respectively, as was determined by GC-MS. The obtained ATR-FTIR spectra for the LEOs indicated as main components the secondary alcohols (linalool and terpinene-4-ol) in LEO *Var.* Moldoveanca 4 from 2018 and the ester compounds (linalyl acetate, lavandulyl acetate) in the other analyzed LEOs samples. The main components influenced the features of the vibrational spectra and the relative absorbance values. The FTIR results are in good agreement with GS-MS data of investigated LEOs samples. The analyzed LEO are rich in linalyl acetate and linalool and present a small amount of camphor, those values being compatible with the legislation of international standards. The studied LEO have good antioxidant activity, as determined by ABTS and DPPH assays. The antimicrobial activity of LEOs against *E. coli, S. aureus*, and *C. albicans* was evaluated and *var*. Vis Magis was the most potent from the LEO tested. None of the LEOs was potent against *P. aeruginosa*. The results obtained indicate that tea tree and geranium essential oils added to LEO could improve the antibacterial effect of *L. angustifolia* essential oil.

## Figures and Tables

**Table 1:** Chemical composition of the 3 varieties of *L. angustifolia*: a. Moldoveanca 4, b. Alba 7, and c. Vis magic 10 essential oils, obtained in 3 consequtive years: 2017, 2018, 2019.

Compound	RT (Min)	*Var.* Moldoveanca 4 (1)			*Var.* Alba 7 (2)			*Var.* Vis Magic 10 (3)		
		2017	2018	2019	2017	2018	2019	2017	2018	2019
(-)-β-bourbonene	24.48	0.11 ± 0.00	0.14 ± 0.01	0.09 ± 0.01	0.09 ± 0.00	0.08 ± 0.00	0.08 ± 0.00	0.08 ± 0.00	0.09 ± 0.01	0.07 ± 0.01
1.8-cineole	8.86	1.46 ± 0.05	3.15 ± 0.12	2.72 ± 0.03	2.19 ± 0.06	1.41 ± 0.03	2.82 ± 0.09	0.77 ± 0.01	0.95 ± 0.12	2.54 ± 0.12
3-carene	8.01	0.21 ± 0.01	1.24 ± 0.01	0.26 ± 0.00	0.55 ± 0.01	0.35 ± 0.01	0.77 ± 0.03	0.20 ± 0.02	0.21 ± 0.02	0.66 ± 0.03
8-hidroxylinalool	23.21	0.28 ± 0.00	0.00 ± 0.00	0.06 ± 0.01	0.08 ± 0.01	0.00 ± 0.00	0.04 ± 0.01	0.05 ± 0.00	0.00 ± 0.00	0.03 ± 0.00
acetic acid. hexyl ester	7.76	0.17 ± 0.01	0.26 ± 0.01	0.18 ± 0.01	0.22 ± 0.02	0.18 ± 0.00	0.34 ± 0.02	0.10 ± 0.01	0.14 ± 0.02	0.35 ± 0.03
bornyl acetate	21.11	0.29 ± 0.01	0.08 ± 0.02	0.38 ± 0.02	0.38 ± 0.03	0.22 ± 0.00	0.46 ± 0.02	0.32 ± 0.07	0.27 ± 0.03	0.43 ± 0.02
butanoic acid. hexyl ester	17.72	0.51 ± 0.01	0.91 ± 0.01	0.61 ± 0.01	0.48 ± 0.01	0.26 ± 0.11	1.01 ± 0.08	0.31 ± 0.02	0.24 ± 0.07	1.08 ± 0.01
camphene	5.57	0.20 ± 0.01	0.31 ± 0.00	0.35 ± 0.01	0.38 ± 0.01	0.21 ± 0.01	0.42 ± 0.01	0.19 ± 0.01	0.19 ± 0.00	0.33 ±0.02
camphor	15.19	0.43 ± 0.06	0.19 ± 0.02	0.13 ± 0.02	0.49 ± 0.01	0.36 ± 0.00	0.98 ± 0.05	0.39 ± 0.01	0.41 ± 0.06	0.12 ± 0.04
caryophyllene	25.49	4.47 ± 0.06	5.39 ± 0.20	5.41 ± 0.02	4.26 ± 0.13	3.55 ± 0.14	4.53 ± 0.00	3.95 ± 0.05	4.10 ± 0.15	4.58 ± 0.03
caryophyllene oxide	29.67	0.68 ± 0.02	0.46 ± 0.02	0.17 ± 0.00	0.31 ± 0.01	0.23 ± 0.01	0.42 ± 0.01	0.28 ± 0.01	0.46 ± 0.09	0.23 ± 0.00
*cis*-α-bergamotene	25.92	0.24 ± 0.01	0.55 ± 0.02	0.30 ± 0.04	0.26 ± 0.02	0.22 ± 0.04	0.26 ± 0.03	0.23 ± 0.01	0.23 ± 0.01	0.20 ± 0.03
*cis*-geranyl acetate	24.17	0.21 ± 0.00	0.29 ± 0.01	0.14 ± 0.01	0.56 ± 0.02	0.57 ± 0.02	0.60 ± 0.01	0.52 ± 0.01	0.47 ± 0.02	0.46 ± 0.01
*cis*-linalool oxide	11.75	0.44 ± 0.03	0.52 ± 0.01	0.27 ± 0.01	0.36 ± 0.02	0.36 ± 0.01	0.29 ± 0.01	0.29 ± 0.02	0.35 ± 0.03	0.19 ± 0.02
*cis*-β ocimene	10.19	1.36 ± 0.04	0.43 ± 0.01	2.70 ± 0.00	2.08 ± 0.01	2.11 ± 0.01	2.27 ± 0.04	2.15 ± 0.10	1.99 ± 0.03	2.83 ± 0.08
D-limonene	8.91	0.69 ± 0.02	2.85 ± 0.12	1.66 ± 0.03	2.06 ± 0.11	1.54 ± 0.03	3.12 ± 0.06	1.00 ± 0.07	0.94 ± 0.10	2.58 ± 0.07
germacrene D	27.14	1.08 ± 0.02	1.01 ± 0.02	1.16 ± 0.01	0.86 ± 0.03	1.31 ± 0.05	0.81 ± 0.01	1.27 ± 0.03	1.27 ± 0.05	0.86 ± 0.03
isocaryophyllene	26.43	2.74 ± 0.05	2.72 ± 0.10	2.92 ± 0.01	2.37 ± 0.06	2.98 ± 0.11	2.27 ± 0.01	3.16 ± 0.05	3.06 ± 0.08	2.39 ± 0.02
lavandulyl acetate	21.36	2.09 ± 0.03	0.34 ± 0.02	3.11 ± 0.01	2.45 ± 0.04	1.51 ± 0.04	2.55 ± 0.00	1.79 ± 0.04	1.94 ± 0.11	2.70 ± 0.06
linalool	14.17	33.27 ± 0.24	46.83 ± 0.84	32.19 ± 0.02	31.97 ± 0.35	32.26 ± 0.24	33.77 ± 0.19	29.93 ± 0.22	30.97 ± 0.51	34.61 ± 0.12
linalyl acetate	20.40	35.18 ± 0.53	17.70 ± 0.29	31.45 ± 0.03	33.42 ± 0.04	35.32 ± 0.22	28.03 ± 0.10	37.13 ± 0.84	36.80 ± 0.63	27.55 ± 0.39
linalyl formate	23.62	0.11 ± 0.00	0.15 ± 0.01	0.10 ± 0.01	0.25 ± 0.00	0.29 ± 0.01	0.27 ± 0.01	0.26 ± 0.00	0.24 ± 0.01	0.58 ± 0.02
m-cymene	8.43	0.02 ± 0.00	0.02 ± 0.00	0.00 ± 0.00	0.03 ± 0.00	0.01 ± 0.00	0.04 ± 0.01	0.01 ± 0.00	0.01 ± 0.00	0.04 ± 0.02
p-cymene	8.63	0.21 ± 0.01	0.24 ± 0.00	0.05 ± 0.00	0.19 ± 0.01	0.14 ± 0.01	0.09 ± 0.02	0.13 ± 0.01	0.15 ± 0.01	0.06 ± 0.02
tau-cadinol	31.27	0.14 ± 0.01	0.10 ± 0.01	0.04 ± 0.00	0.10 ± 0.00	0.05 ± 0.00	0.29 ± 0.00	0.07 ± 0.00	0.08 ± 0.01	0.13 ± 0.01
terpinen-4-ol	17.45	6.71 ± 0.06	7.70 ± 0.12	3.63 ± 0.14	4.26 ± 0.05	6.53 ± 0.04	3.10 ± 0.04	7.16 ± 0.14	6.32 ± 0.36	3.06 ± 0.13
trans-linalool oxide	12.70	0.27 ± 0.01	0.53 ± 0.01	0.14 ± 0.00	0.39 ± 0.00	0.33 ± 0.01	0.28 ± 0.00	0.24 ± 0.02	0.26 ± 0.03	0.21 ± 0.01
trans-β-ocimene	9.50	3.17 ± 0.09	1.22 ± 0.01	6.99 ± 0.01	4.56 ± 0.02	3.89 ± 0.01	5.56 ± 0.03	4.19 ± 0.16	4.07 ± 0.04	6.66 ± 0.06
α cedrene	25.33	0.10 ± 0.00	0.19 ± 0.01	0.08 ± 0.00	0.10 ± 0.00	0.08 ± 0.00	0.09 ± 0.00	0.09 ± 0.00	0.08 ± 0.00	0.07 ± 0.00
α limonene diepoxide	23.31	0.17 ± 0.00	0.00 ± 0.00	0.06 ± 0.01	0.05 ± 0.00	0.00 ± 0.00	0.06 ± 0.01	0.03 ± 0.00	0.00 ± 0.00	0.03 ± 0.01
α pinene	5.15	0.34 ± 0.01	0.46 ± 0.01	0.30 ± 0.01	0.39 ± 0.00	0.34 ± 0.00	0.37 ± 0.01	0.38 ± 0.02	0.36 ± 0.00	0.32 ± 0.01
α thujene	4.94	0.14 ± 0.01	0.26 ± 0.01	0.09 ± 0.01	0.13 ± 0.00	0.15 ± 0.00	0.14 ± 0.00	0.17 ± 0.01	0.15 ± 0.00	0.12 ± 0.01
α-santoline alcohol	17.22	1.11 ± 0.01	1.29 ± 0.01	0.96 ± 0.04	1.59 ± 0.00	0.90 ± 0.01	0.71 ± 0.01	1.12 ± 0.02	1.05 ± 0.02	0.73 ± 0.02
α-terpineol	18.23	0.61 ± 0.00	0.96 ± 0.02	0.09 ± 0.00	0.85 ± 0.02	1.02 ± 0.01	1.05 ± 0.00	0.98 ± 0.02	0.92 ± 0.02	1.14 ± 0.04
β myrcene	7.07	0.26 ± 0.01	0.79 ± 0.05	0.54 ± 0.02	0.53 ± 0.01	0.62 ± 0.00	1.01 ± 0.01	0.54 ± 0.02	0.53 ± 0.03	1.00 ± 0.02
β-pinene	6.51	0.21 ± 0.01	0.22 ± 0.01	0.29 ± 0.01	0.28 ± 0.00	0.17 ± 0.01	0.46 ± 0.01	0.09 ± 0.01	0.15 ± 0.03	0.47 ± 0.02
γ-cadinene	27.99	0.19 ± 0.01	0.23 ± 0.01	0.18 ± 0.02	0.33 ± 0.01	0.16 ± 0.01	0.47 ± 0.03	0.20 ± 0.00	0.17 ± 0.01	0.33 ± 0.01
γ-terpinene	10.83	0.13 ± 0.00	0.24 ± 0.00	0.08 ± 0.00	0.15 ± 0.00	0.21 ± 0.01	0.15 ± 0.01	0.23 ± 0.02	0.21 ± 0.01	0.19 ± 0.02

**Table 2:** The ATR-FTIR absorption band for LEO samples and vibrations assignments.

Wavenumbers (cm^−1^) of ATR-FTIR Absorption Band for LEOs									Vibration Assignment	Ref.
*Var.* Moldoveanca 4 (1)			*Var.* Alba 7 (2)			*Var.* Vis Magic 10 (3)				
2017	2018	2019	2017	2018	2019	2017	2018	2019		
3455	3454	3457	3455	3456	3454	3455	3453	3457	ν (O–H) from alcohols	[22]
3088	3086	3088	3088	3088	3088	3088	3088	3088	ν (=C–H, Csp^2^); ν_asym_ (C-H) and ν_sym_ (C–H) from CH_3_ group; ν_asym_ (C–H) and ν_sym_ (C–H) from CH_2_ group.	[22,23]
2968	2967	2968	2968	2968	2968	2968	2968	2968		
2929	2925	2925	2925	2925	2925	2925	2925	2925		
2878	2877	2877	2879	2879	2875	2879	2879	2876		
2858	2858	2858	2859	2858	2858	2585	2858	2858		
2730	2730	2730	2729	2730	2730	2730	2730	2730		
1738	1738	1738	1738	1738	1738	1737	1737	1738	ν (C=O) carbonyl group from aliphatic esters; ν (C=C–C) alkyl group from alkenes; ν (C=C) from unsaturated compouns.	[22,24]
nd	1723	nd	nd	nd	nd	nd	nd	nd		
1674	1673	1674	1674	1674	1674	1674	1674	1674		
1644	1643	1644	1644	1644	1644	1644	1644	1644		
1595	1596	1595	1596	1595	1596	1595	1595	1595		
1450	1450	1450	1450	1450	1450	1450	1450	1450	δ _asym_ (C–H) and (C–H) in-plane bending from CH_3_ and CH_2_ groups.	[22,25,26]
1412	1411	1412	1412	1412	1412	1412	1412	1412		
1369	1373	1370	1370	1370	1370	1369	1369	1371		
1239	1239	1239	1239	1239	1239	1239	1239	1239	ν _asym_ (C–O) and ν _sym_ (C–O) from ester group; ν_asym_ (C–O) and ν_sym_ (C–O) from alcohols; δ _sym_ (CH_3_(CO)), ν_asym_ (C–O–C) and ν_sym_ (C–O–C); δ (O-H) in-plane from secondary alcohols.	[22,24,25,27]
nd	1207	nd	nd	nd	nd	nd	nd	nd		
1171	1171	1171	1171	1171	1171	1171	1171	1171		
1110	1111	1109	1110	1110	1109	1109	1110	1109		
1092	1091	1093	1093	1093	1093	1092	1092	1093		
1018	1016	1018	1018	1018	1018	1018	1018	1018		
994	993	993	993	994	993	993	993	993	δ (C–H), ω (CH_2_), ω (C–H) out-of-plane; ω (O–H) out-of-plane from alcohols.	[22,23,28]
919	919	919	919	919	919	919	919	919		
863	864	863	863	863	863	863	863	863		
834	835	834	834	834	834	834	834	834		
738	736	740	739	739	738	739	739	738		
690	689	690	690	690	690	690	690	690		

Legend: nd—not detected; vibrations: ν—stretching; δ—bending; ω—wagging; asym—asymmetric deformation; sym—symmetric deformation.

**Table 3:** The antioxidant activity determined by ABTS and DPPH assays for the essential oils obtained in 3 years (2017–2019) for *Lavandula angustifolia* (Moldoveanca 4, Alba 7, and Vis magic 10), where *p* < 0.05.

Essential Oil	Year	ABTS Assay		DPPH Assay	
		Inhibition%	mmol TEAC/L	Inhibition%	mg Trolox/L
*Var.* Moldoveanca 4 (1)	2017	66.15 ± 4.99 ^a^	0.5959 ± 0.0577	52.66 ± 4.09 ^a^	1.8364 ± 0.2209
	2018	56.23 ± 12.77 ^a^	0.4811 ± 0.1478	24.10 ± 4.88 ^b^	1.8369 ± 0.2638
	2019	57.19 ± 3.14 ^a^	0.4923 ± 0.0363	42.70 ± 0.24 ^c^	1.2980 ± 0.0129
*Var.* Alba 7 (2)	2017	71.36 ± 4.64 ^a^	0.6562 ± 0.0536	61.57 ± 2.51 ^a^	2.3184 ± 0.1359
	2018	62.23 ± 8.43 ^a^	0.5506 ± 0.0975	58.68 ± 2.19 ^a^	2.1620 ± 0.1182
	2019	56.20 ± 2.79 ^a^	0.4808 ± 0.0322	32.37 ± 2.35 ^b^	0.7393 ± 0.1271
*Var.* Vis magic 10 (3)	2017	65.29 ± 6.06 ^a^	0.5859 ± 0.0701	69.83 ± 8.23 ^a^	2.7654 ± 0.4452
	2018	76.74 ± 4.15 ^a^	0.7185 ± 0.0480	76.17 ± 4.18 ^a^	3.1080 ± 0.2261
	2019	72.04 ± 5.78 ^a^	0.6640 ± 0.0668	39.39 ± 1.19 ^b^	1.1192 ± 0.0645

TEAC—Trolox equivalent antioxidant capacities. The results are shown as mean ± standard deviation and superscript different letters (a, b, c) denote significant differences between data for a species harvested in three different years (2017, 2018, 2019), for one test, by applying Tukey’s test for *p* < 0.05. Means with superscripts bearing the same letter in the data from a species are not significantly different.

**Table 4:** Diameters of the inhibition zones of EOs against *S. aureus*.

No. of the Sample	Essential Oil	Inhibition Zone (mm)	
		ATCC	Clinical Isolates
Single			
1	*Var.* Moldoveanca 4	18.5 ± 2.12	18.0 ± 0.00
2	*Var.* Alba 7	19.5 ± 0.70	18.5 ± 2.12
3	*Var.* Vis magic 10	21.5 ± 0.70	20.0 ± 0.70
In combination with geranium or tea tree EO (*v*/*v*)			
4	*Var.* Alba 7:EO geranium = 1:1	24.5 ± 0.70	25.0 ± 1.00
5	*Var.* Alba 7: EO geranium = 2:1	27.0 ± 1.41	27.0 ± 0.70
6	*Var.* Alba 7: EO tea tree = 1:1	31.0 ± 1.12	33.5 ± 0.70
7	*Var.* Alba 7:EO tea tree = 2:1	30.0 ± 1.00	31.0 ± 1.12

Controls: Vancomycin: 21.5 ± 0.71 mm, Eritromycin: 27 ± 1.41 mm, Clindamycin: 27.5 ± 0.71 mm, Moxifloxacin: 29.5 ± 2.12 mm.

**Table 5:** Diameters of the inhibition zones of EOs against *E. coli*.

No. of the Sample	Essential Oil	Inhibition Zone (mm)	
		ATCC	Clinical Isolates
Single			
1	*Var.* Moldoveanca 4	18.0 ± 2.12	19.5 ± 0.70
2	*Var.* Alba 7	18.5 ± 2.12	18.5 ± 2.12
3	*Var.* Vis magic 10	25.5 ± 0.70	24.5 ± 0.70
In combination with geranium or tea tree EO (*v*/*v*)			
4	*Var.* Alba 7:EO geranium = 1:1	18.5 ± 0.70	19.5 ± 0.70
5	*Var.* Alba 7: EO geranium = 2:1	22.5 ± 0.70	24.5 ± 0.70
6	*Var.* Alba 7: EO tea tree = 1:1	TI	TI
7	*Var.* Alba 7:EO tea tree = 2:1	TI	TI

Legend: TI–total inhibition; Controls: Ceftazidime: 27.5 ± 0.71 mm, Levofloxacin = 31 ± 0.71 mm, Moxifloxacin: 33.5 ± 2.12 mm.

**Table 6:** Diameters of the inhibition zones of EOs against *C. albicans*.

No. of the Sample	Essential Oil	Inhibition Zone (mm)	
		ATCC	Clinical Isolates
Single			
1	*Var.* Moldoveanca 4	12.5 ± 0.70	13.5 ± 0.70
2	*Var.* Alba 7	13.5 ± 0.70	14.5 ± 0.70
3	*Var.* Vis magic 10	14.5 ± 0.70	14.5 ± 0.70
In combination with geranium or tea tree EO (*v*/*v*)			
4	*Var.* Alba 7:EO geranium = 1:1	TI	TI
5	*Var.* Alba 7: EO geranium = 2:1	TI	TI
6	*Var.* Alba 7: EO tea tree = 1:1	TI	TI
7	*Var.* Alba 7:EO tea tree = 2:1	TI	TI

Legend: TI–total inhibition; Fluconazole: 18.5 ± 2.12 mm, Ketoconazol: 25.5 ± 0.70 mm.

**Table 7:** Minimum inhibitory concentration (MIC) values of EOs against *S. aureus, E. coli,* and *C. albicans*.

No. of the Sample	Essential Oil	MIC (*v*/*v*) %					
		*S. aureus*		*E. coli*		*C. albicans*	
		ATCC	Clinical Isolates	ATCC	Clinical Isolates	ATCC	Clinical Isolates
1	*Var.* Moldoveanca 4	12.5	12.5	25	25	12.5	12.5
2	*Var.* Alba 7	6.25	12.5	12.5	25	6.25	12.5
3	*Var.* Vis magic 10	3.12	3.12	12.5	6.25	3.12	6.25
4	*Var.* Alba 7:EO geranium = 1:1	3.12	3.12	25	25	1.56	3.12
5	*Var.* Alba 7: EO geranium = 2:1	1.56	3.12	6.25	12.5	0.78	1.56
6	*Var.* Alba 7: EO tea tree = 1:1	1.56	1.56	3.12	3.12	0.78	0.78
7	*Var.* Alba 7:EO tea tree = 2:1	1.56	1.56	1.56	1.56	0.78	0.78

**Table 8:** Antibiotic disks used, contents, and reference range according to CLSI 2020.

Strain	Antibiotic (Abbreviation)	Microcompresses Content (µg)	Reference Range
*Staphylococcus aureus* ATCC 25923	Clindamycin (DA)	2	24–30
	Vancomycin (VA)	30	17–21
*Escherichia coli* ATCC 25922	Ceftazidime	30	25–32
	Moxifloxacin	5	28–35
*Pseudomonas aeruginosa* ATCC 27853	Ceftazidime	30	22–29
	Ciprofloxacin	5	25–33
*Candida albicans* ATCC 10231	Fluconazole Ketoconazole	25 15	≥19 ≥28

## Data Availability

The data presented in this study are available in Supplementary Materials.

## Supplementary Materials

The following are available online, Figure S1: Calibration curve for ABTS assay performed with Trolox solutions with different concentrations (0.025–1.0 mM). Figure S2: Calibration curve for DPPH assay performed with Trolox solutions with different concentrations (0.02–4.0 mM).

## Sample Availability

Samples of the compounds are available from the authors.
