# Supplementary material for: Chemical Profile, Antioxidant Capacity, and Antimicrobial Activity of Essential Oils Extracted from Three Different Varieties (Moldoveanca 4, Vis Magic 10, and Alba 7) of Lavandula angustifolia
Source: Molecules. 2021 Jul 20;26(14):4381. doi: 10.3390/molecules26144381 (PMC8303575; doi:10.3390/molecules26144381)
Supplement: Supplementary file 1 [file molecules-26-04381-s001.zip › molecules-1313437-supplementary.pdf]

Supplementary Information

Chemical profile, antioxidant capacity and antimicrobial activity of essential oils extracted from three different varieties (Moldoveanca 4, Vis Magic and Alba 7) of *Lavandula angustifolia*

Mihaela Bogdan[1], Simona Bungau[1,2*], Delia Mirela Tit[1,2], Dana Carmen Zaha[3,4], Aurelia Cristina Nechifor[5], Tapan Behl[6], Dorina Chambre[7,8], Andreea Ioana Lupitu[8], Lucian Copolovici[7,8] and Dana Maria Copolovici[7,8]

The calibration curves calculated for Trolox used in ABTS and DPPH assays, respectively are presented in Figures S1 and S2.

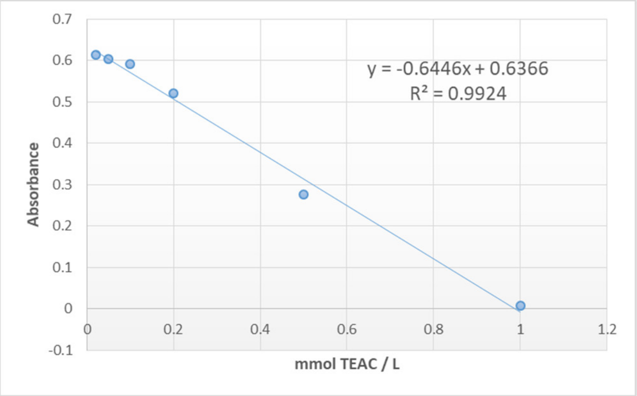

Figure S1. Calibration curve for ABTS assay performed with Trolox solutions with different concentrations (0.025 mM-1.0 mM).

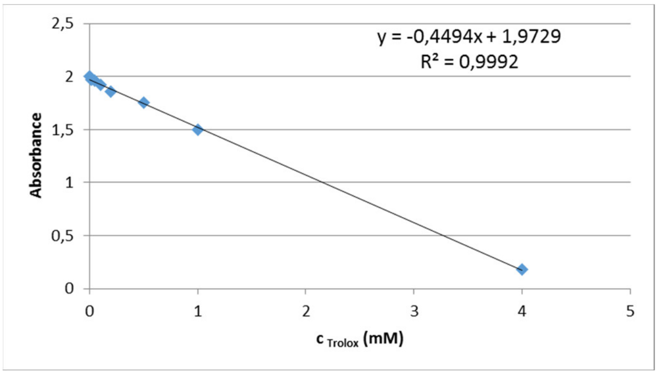

Figure S2. Calibration curve for DPPH assay performed with Trolox solutions with different concentrations (0.02 mM-4.0 mM).
